# Beta emitters rhenium‐188 and lutetium‐177 are equally effective in radioimmunotherapy of HPV‐positive experimental cervical cancer

**DOI:** 10.1002/cam4.562

**Published:** 2015-12-02

**Authors:** Rebecca Phaeton, Zewei Jiang, Ekaterina Revskaya, Darrell R. Fisher, Gary L. Goldberg, Ekaterina Dadachova

**Affiliations:** ^1^ Department of Obstetrics and GynecologyPenn State Hershey Medical CenterHersheyPennsylvania; ^2^ Department of RadiologyAlbert Einstein College of MedicineBronxNew York; ^3^ Dade Moeller Health SystemRichlandWashington; ^4^ Department of Obstetrics and GynecologyAlbert Einstein College of MedicineBronxNew York

**Keywords:** Cervical cancer, E6 and E7 oncogenes, luthetium‐177, radioimmunotherapy, rhenium‐188

## Abstract

Cervical cancer caused by the infection with the human papillomavirus (HPV) remains the fourth leading killer of women worldwide. Therefore, more efficacious treatments are needed. We are developing radioimmunotherapy (RIT) of HPV‐positive cervical cancers by targeting E6 and E7 viral oncoproteins expressed by the cancer cells with the radiolabeled monoclonal antibodies (mAbs). To investigate the influence of different radionuclides on the RIT efficacy—we performed RIT of experimental cervical cancer with Rhenium‐188 (^188^Re) and Lutetium‐177 (^177^Lu)‐labeled mAb C1P5 to E6. The biodistribution of ^188^Re‐ and ^177^Lu‐labeled C1P5 was performed in nude female mice bearing CasKi cervical cancer xenografts and the radiation dosimetry calculations for the tumors and organs were carried out. For RIT the mice were treated with 7.4 MBq of either ^188^Re‐C1P5 or ^177^Lu‐C1P5 or left untreated, and observed for their tumor size for 28 days. The levels of ^188^Re‐ and ^177^Lu‐C1P5 mAbs‐induced double‐strand breaks in CasKi tumors were compared on days 5 and 10 post treatment by staining with anti‐gamma H2AX antibody. The radiation doses to the heart and lungs were similar for both ^177^Lu‐C1P5 and ^188^Re‐C1P5. The dose to the liver was five times higher for ^177^Lu‐C1P5. The doses to the tumor were 259 and 181 cGy for ^177^Lu‐C1P5 and ^188^Re‐C1P5, respectively. RIT with either ^177^Lu‐C1P5 or ^188^Re‐C1P5 was equally effective in inhibiting tumor growth when each was compared to the untreated controls (*P* = 0.001). On day 5 there was a pronounced staining for gamma H2AX foci in ^177^Lu‐C1P5 group only and on day 10 it was observed in both ^177^Lu‐C1P5 and ^188^Re‐C1P5 groups. ^188^Re‐ and ^177^Lu‐labeled mAbs were equally effective in arresting the growth of CasKi cervical tumors. Thus, both of these radionuclides are candidates for the clinical trials of this approach in patients with advanced, recurrent or metastatic cervical cancer.

## Introduction

In the current era of screening protocols, vaccination strategies and treatment algorithms, infections with human papillomavirus (HPV) should be eradicated. However, annually over 530,000 women are newly diagnosed worldwide with HPV‐related cervical cancer with 88% of cases occurring in developing countries 1. Cervical cancer remains the fourth leading killer of women worldwide and widely fatal in populations who are unable to clear HPV infection, namely patients with human immunodeficiency virus (HIV) 2. At present, when primary chemotherapy and radiation therapy fail, the 5‐year overall survival is reported to be only 3.2–13%. 3 Therefore, more efficacious and targeted treatments are needed. Persistent HPV infections cause not only cervical cancer, but result in a significant percentage of head and neck, penile, anal and vulvar cancers that cumulatively represent 5.2% of the world cancer burden 4.

HPV‐induced cancers express E6 and E7 viral oncogenes which result in lateral expansion and immortalization of infected cells. Both oncogenes act to promote cellular proliferation and inhibit apoptosis; E6 oncogene affects the p53 pathway leading to its rapid degradation via the ubiquitin‐dependent pathway, whereas E7 binds to the retinoblastoma (pRb) gene causing ineffective regulation of cell growth and deregulation of mitosis 5, 6, 7. In our development of the radioimmunotherapy (RIT) approach against HPV‐induced malignancies of head and neck and uterine cervical origin, we targeted E6 and E7 oncoproteins expressed in those cancers with E6‐ or E7‐specific monoclonal antibodies (mAbs) tagged to Rhenium‐188 (^188^Re) radioisotope. Consistent and reproducible tumor growth inhibition was noted in murine models of human cervical and head and neck cancers 8, 9, 10, 11, 12, 13. Both head and neck and cervical cancer are classified as solid tumors, and as they increase in size and outgrow their blood supply, cellular necrosis occurs. The accessibility of intranuclear oncoproteins E6 and E7 to the targeting mAbs is therefore due to the necrosis associated with cellular turnover and release of cellular contents into the interstitial space.

In all our previous RIT studies we used ^188^Re, a high‐energy beta emitter (beta max 2.12 MeV) that exhibits a 3.5 mm average tissue penetration depth and has a short physical half‐life of 16.9 h. In addition, ^188^Re is a nonbone seeking and nonresidualizing radioisotope that does not linger in nontarget organs or blood, making ^188^Re particularly attractive for therapy. When ^188^Re separates from the carrier protein molecule in vivo as a result of catabolism, and oxidizes back to a chemically inert perrhenate anion—it is quickly excreted through the kidneys, leaving little time to cause significant toxicities. To date, ^188^Re has been used in variety of clinical trials 14, 15. During the last decade the RIT armamentarium of radioisotopes has been enriched by the addition of commercially available Lutetium‐177 (^177^Lu), an intermediate energy beta emitter (beta max 0.13 MeV) with 0.7 mm range in tissue and a long physical half‐life of 6.7 days. ^177^Lu demonstrated encouraging results in therapeutic clinical trials especially of somatostatin receptors‐binding radiolabeled peptides 16. Due to its radiolanthanide chemistry, ^177^Lu is a residualizing radioisotope which is excreted primarily via hepatobilliary route. We hypothesize that ^177^Lu‐labeled mAbs could be developed as novel therapeutics for RIT of HPV‐induced malignancies and sought to characterize their potential in a human cervical cancer murine model. In this study, we investigated how the different nuclear decay parameters (penetration range, half‐life, energy of the beta‐emission) and chemistries of ^188^Re and ^177^Lu influence the efficacy and mechanism of RIT targeting HPV‐16/18 E6 oncoprotein in experimental cervical cancer.

## Materials and Methods

### Cell lines, antibodies, and reagents

The commercially available CasKi human cervical cancer cell line, expressing both E6 and E7 oncoproteins, was purchased from the American Type Culture Collection (Manassas, VA). Cells were grown in Roswell Park Memorial Institute Medium (RPMI)‐1640 medium containing 10% fetal bovine serum (FBS) (Sigma) and 1% Penicillin‐streptomycin solution (Sigma, St. Lois, MO, USA, Penicillin 10,000 U and streptomycin 10 mg/mL) at 37°C in a 5% CO_2_ incubator. Matrigel, used in development of tumors, was purchased from BD Biosciences (Rockville, MD). Murine mAb C1P5 (IgG1) to HPV‐16 E6 and HPV‐18 E6 was procured from Abcam, Cambridge, MA, USA.

### Radiolabeling of antibodies

The beta‐emitter ^188^Re (half‐life, 16.9 h) was produced from beta decay of a parent radionuclide ^188^W (half‐life 69 days) using a ^188^W/^188^Re generator (ITG Isotope Technologies Garching GmbH, Garching, Germany). ^177^Lu was purchased from PerkinElmer. After ^188^Re was eluted from a generator in the form of sodium perrhenate, it was reduced to the lower oxidation states with tin (II) chloride, and the antibodies were labeled with ^188^Re “directly” through binding of reduced ^188^Re to the generated sulfhydryl groups on the antibodies 17. For radiolabeling with ^177^Lu, C1P5 mAb was first conjugated to the bifunctional chelating agent p‐SCN‐Bn‐DOTA (Macrocyclics, Dallas, TX) as in 18 and then radiolabeled with ^177^Lu as in 19. The number of DOTA molecules conjugated to a C1P5 antibody molecule was determined to be 1.0 ± 0.3 by the spectrophotometric assay as in 20. The radiolabeling yields for ^188^Re‐ and ^177^Lu‐labeled C1P5 mAb were measured by instant thin layer chromatography (ITLC) by developing silica gel (SG) 10 cm strips in saline. In this system the radiolabeled antibodies stay at the point of application while free ^188^Re and ^177^Lu in form of EDTA – ethylenediaminetetraacetic acid; complex move with the solvent front. The average radiolabeling yield for either ^188^Re‐ or ^177^Lu‐C1P5 mAb was 85 ± 5%. The radiolabeled mAbs were purified by high performance liquid chromatography (HPLC) using TosoHaas size exclusion column with phosphate buffered saline (PBS) at 1 mL/min as an eluent using Waters high performance liquid chromatography (HPLC) system equipped with UV and radiation (Bioscan, Poway, CA, USA) flow‐through detectors.

### HPV tumor model

All animal studies were approved by the Institute for Animal Studies at the Albert Einstein College of Medicine. Twenty‐five six‐week‐old female athymic balb/c nude mice were purchased from Charles River Laboratories, Wilmington, MA, USA. 8 × 10^6^ CasKi cells mixed with Matrigel were implanted into the flanks of mice in a subcutaneous fashion and allowed to grow to tumor size of 3–5 mm before randomization into the biodistribution or treatment groups.

### Biodistribution of ^188^Re‐C1P5 and ^**177**^Lu‐C1P5 mAbs in tumor‐bearing mice and dosimetry calculations

For biodistribution experiments, the CasKi tumor‐bearing mice were randomized into the groups of four mice per group and injected with a single intraperitoneal (IP) dose of 1.85 MBq ^188^Re‐C1P5 or ^177^Lu‐C1P5. At the predetermined time points of 24, 48, and 72 h the group of ^188^Re‐C1P5‐injected mice and the group of ^177^Lu‐C1P5‐injected mice were killed, their tumors and major organs removed, blotted from blood, weighed, counted for radioactivity in a gamma counter, and percentage of injected dose per gram (ID/g) organ/tissue was calculated. These radioactive decay‐corrected data were used to evaluate antibody biodistribution (uptake and clearance kinetics) for radiation dosimetry calculations. Each mouse organ or tissue has a uniquely calculated absorbed fraction depending on geometry (and density of tissue, bone, and lungs) and on complete energy spectrum of the applicable radionuclide. The electron energy absorbed fractions in mouse organs or tissues for ^188^Re vary from 0.172 to 0.709 and from 0.88 to 0.99 for ^177^Lu 21. The effective biodistribution data for each organ and time point were plotted and then fit by least‐squares linear regression to an exponential function. The correlation coefficients are given in the last column of Table 1 under the heading “*r* ^2^”. The areas under curve were then integrated, multiplied by the equilibrium dose constant and absorbed fraction, and corrected for units. The equilibrium dose constants for ^188^Re and ^177^Lu were obtained from the updated radionuclide emission data provided in 22. The equilibrium dose constant was determined only for electrons because only negligible photon radiation is absorbed by the small mouse organs. The results were expressed in cGy per 37 Bq.

**Table 1:** Dosimetry results for ^188^Re‐C1P5 and ^177^Lu‐C1P5 monoclonal antibodies administered to CasKi tumor‐bearing mice

Organ	cGy/37 Bq	Label	*r* ^2^
Blood	5.292	^188^‐Re	0.98
Lung	1.066	^188^‐Re	0.99
Heart	1.633	^188^‐Re	0.98
Spleen	0.903	^188^‐Re	0.99
Liver	1.400	^188^‐Re	0.98
Kidney	2.138	^188^‐Re	0.99
Stomach	1.071	^188^‐Re	0.99
Tumor	0.907	^188^‐Re	0.99
Muscle	0.712	^188^‐Re	0.96
Bone	0.698	^188^‐Re	0.98

Organ	cGy/37 Bq	Label	*r* ^2^
Blood	6.036	^177^‐Lu	0.99
Lung	1.081	^177^‐Lu	0.91
Heart	1.678	^177^‐Lu	0.99
Spleen	1.774	^177^‐Lu	0.99
Liver	7.919	^177^‐Lu	0.71
Kidney	1.540	^177^‐Lu	0.95
Stomach	0.481	^177^‐Lu	0.97
Tumor	1.297	^177^‐Lu	0.70
Muscle	0.317	^177^‐Lu	0.86
Bone	0.450	^177^‐Lu	0.87

### RIT of CasKi tumor‐bearing mice with ^188^Re‐ and ^177^Lu‐labeled C1P5 mAbs

CasKi tumor‐bearing mice were randomized into three groups of five animals each: untreated controls, 7.4 MBq ^188^Re‐C1P5 mAb and 7.4 MBq ^177^Lu‐C1P5 mAb. Therapy was administered as a 200 *μ*L single IP injection. The total amount of an antibody per mouse in both ^188^Re‐ and ^177^Lu‐C1P5 mAbs groups was 10 *μ*g. The mice were observed for 28 days and the tumors were measured in three dimensions using electronic calipers every 3 days during the observation period. The volume of the tumors was calculated in the following fashion: *V* = (*x* × *y* × *z*)/2. The experiment was performed two times.

### Analysis of radiation‐induced DNA damage during RIT

In a separate experiment, we compared the levels of radiation‐induced double‐strand breaks (DSB) for ^188^Re‐ and ^177^Lu‐C1P5 mAbs. CasKi tumor‐bearing mice (six animals per group) were treated with the same doses of ^188^Re‐ and ^177^Lu‐C1P5 mAbs or left untreated as in RIT experiment described above. Three mice out of each group were killed on day 5 and the remaining three mice—on day 10 post treatment, the tumors were removed, fixed, cut into 5‐*μ*m‐thick sections, and stained with the anti‐gamma H2AX antibody (Novus Biologicals, Littleton, CO, USA, cat. #NB100‐384) which detects phosphorylated histone 2AX by incubating the slides with the 1:2000 dilution of the antibody overnight at 4°C. The stained slides were subsequently incubated for 1 h at room temperature with the goat anti‐rabbit IgG Dylight 594 secondary antibody (Thermo Scientific, Waltham, MA, USA, cat. #35560) at 1:100 dilution and mounted with the VECTASHIELD mounting medium with 4’6‐diamidino‐2‐phenylindole (DAPI) (Vector Laboratories, Burlingame, CA, USA, cat# H‐1200).

### Statistical analyses

Power was estimated using PASS version 11 (NCSS, Inc., Kaysville, UT, http://www.ncss.com) using simulations of different tumor volumes based on our previously published data with the radiolabeled C1P5 mAb 8, 9, 10, 11, 12, 13 and conservative assumptions regarding the groups treated with the radiolabeled mAbs. All simulations showed power of at least 83% with only five animals per group because of the large differences between treated and untreated animals. Based on this power estimation, five mice per group were utilized in therapy studies and six mice per group—in the DNA damage study. In addition, therapy experiments were performed twice. The differences between the groups were analyzed using Kruskal–Wallis test.

## Results

### Biodistribution demonstrated differential uptake patterns and clearance times of ^188^Re‐ and ^177^Lu‐C1P5 mAbs in the tumors and some normal organs

**Figure 1:**
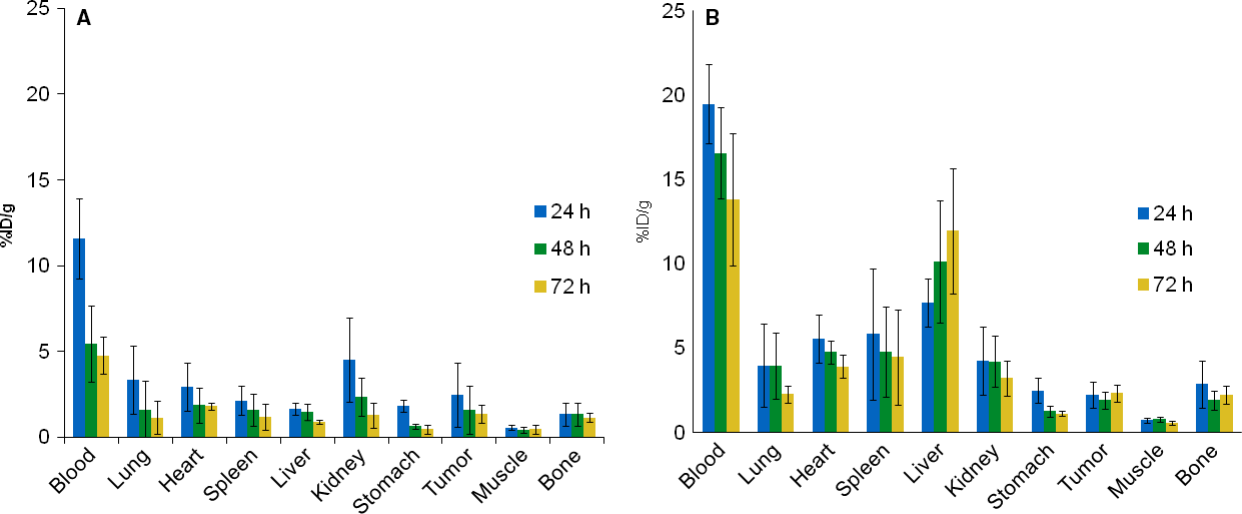
Biodistribution of ^188^Re‐C1P5 and ^177^Lu‐C1P5 monoclonal antibodies (mAbs) in CasKi tumor‐bearing mice: (A) ^188^Re‐C1P5; (B) ^177^Lu‐C1P5 mAbs. The mice were injected intraperitoneal with 1.85 MBq of ^188^Re‐C1P5 and ^177^Lu‐C1P5 mAbs and killed at 24, 48, and 72 h for tissue harvesting. Four mice per group were used.

We started our comparative study of ^188^Re‐ and ^177^Lu‐C1P5 mAbs with the biodistribution in CasKi tumor‐bearing mice. Figure 1 shows that the uptake of ^177^Lu‐C1P5 mAb in the blood, lungs, heart, spleen, liver, and bone was higher than that of ^188^Re‐C1P5 at the 24 h time point and was also clearing significantly slower at 48 and 72 h than the uptake noted for ^188^Re‐C1P5. The uptake in the kidneys at 24 h was the same for both antibodies but ^188^Re‐C1P5 cleared much faster at the later time points. The uptake and clearance rates noted in stomach and muscle were very close for both antibodies. The uptake of both ^177^Lu‐C1P5 and ^188^Re‐C1P5 in the tumor was ~2.5% ID/g at 24 h. However, over time, at 48 and 72 h, the uptake of ^188^Re‐C1P5 in the tumor decreased, while the uptake of ^177^Lu‐C1P5 remained the same.

### The doses to the tumor for ^177^Lu‐C1P5 and ^188^Re‐C1P5 were close to each other

The effective biodistribution data for each organ and time point were plotted and then fit by least‐squares linear regression to an exponential function resulting in high correlation coefficients (Table 1, last column). Dosimetry calculations demonstrated that the doses to the blood and blood‐rich organs such as heart and lungs were remarkably similar for both ^177^Lu‐C1P5 and ^188^Re‐C1P5 (Table 1). The dose to the spleen was approximately two times higher for ^177^Lu‐C1P5 and the doses to the stomach and muscle were approximately two times higher for ^188^Re‐C1P5. The most pronounced difference was seen in the dose to the liver that was approximately five times higher for ^177^Lu‐C1P5. The dose to the tumor was 1.4 times higher for ^177^Lu‐C1P5—259 and 181 cGy for ^177^Lu‐C1P5 and ^188^Re‐C1P5, respectively.

### ^177^Lu‐C1P5 and ^188^Re‐C1P5 exhibited equal efficacy of tumor inhibition in CasKi tumor‐bearing mice

**Figure 2:**
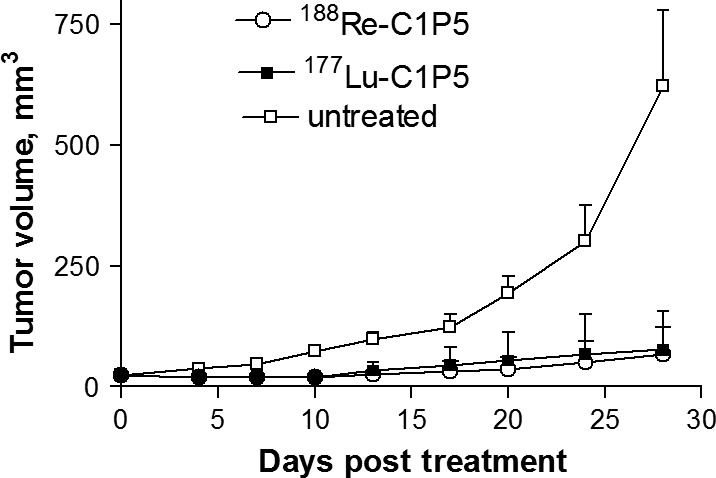
Radioimmunotherapy of CasKi tumor‐bearing mice with ^188^Re‐C1P5 and ^177^Lu‐C1P5 monoclonal antibodies (mAbs). Groups of 5 mice received 7.4 MBq of either ^188^Re‐C1P5 or ^177^Lu‐C1P5 mAbs or were left untreated. The experiment was performed twice.

The tumor volumes in mice treated with ^177^Lu‐C1P5 or ^188^Re‐C1P5 are shown in Figure 2. CasKi tumors grew aggressively and all untreated mice had to be killed on Day 28 post‐RIT administration. RIT with either ^177^Lu‐C1P5 or ^188^Re‐C1P5 was an equally effective inhibitor of tumor growth and statistically significant (*P* = 0.001) in comparison to the untreated controls. There was no difference between ^177^Lu‐C1P5 and ^188^Re‐C1P5 in their effect on the tumor (*P* = 0.1).

### ^177^Lu‐C1P5 and ^188^Re‐C1P5 demonstrated differential timing of radiation damage to the DNA

**Figure 3:**
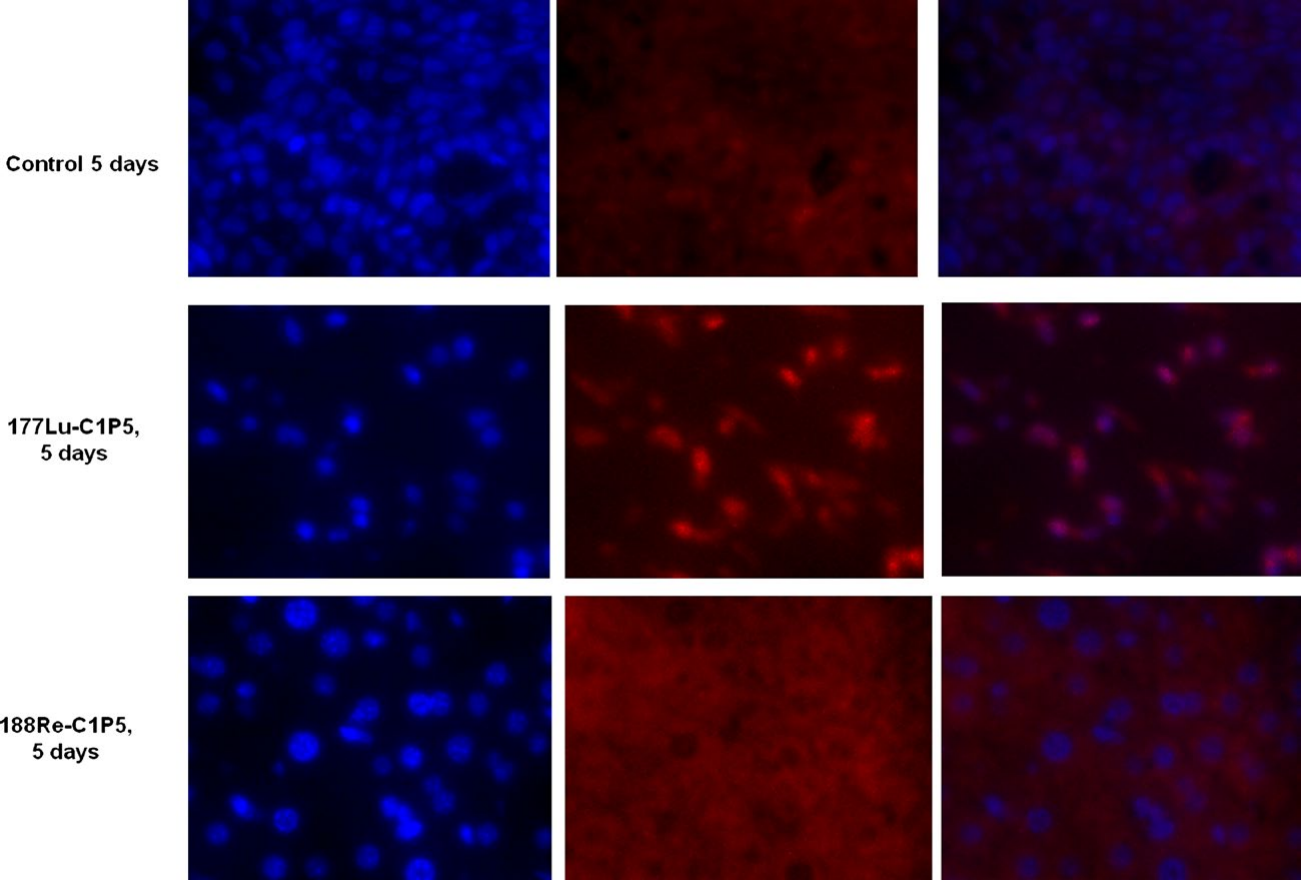
Immunohistochemistry staining for gamma H2AX foci in ^188^Re‐C1P5 and ^177^Lu‐C1P5 tumors on Day 5 post radioimmunotherapy. First row—untreated controls, second row—^177^Lu‐C1P5, third row—^188^Re‐C1P5 treatment. First column—DAPI staining (blue), second column—gamma H2AX foci staining (red), third column—superimposition of DAPI and gamma H2AX staining.

**Figure 4:**
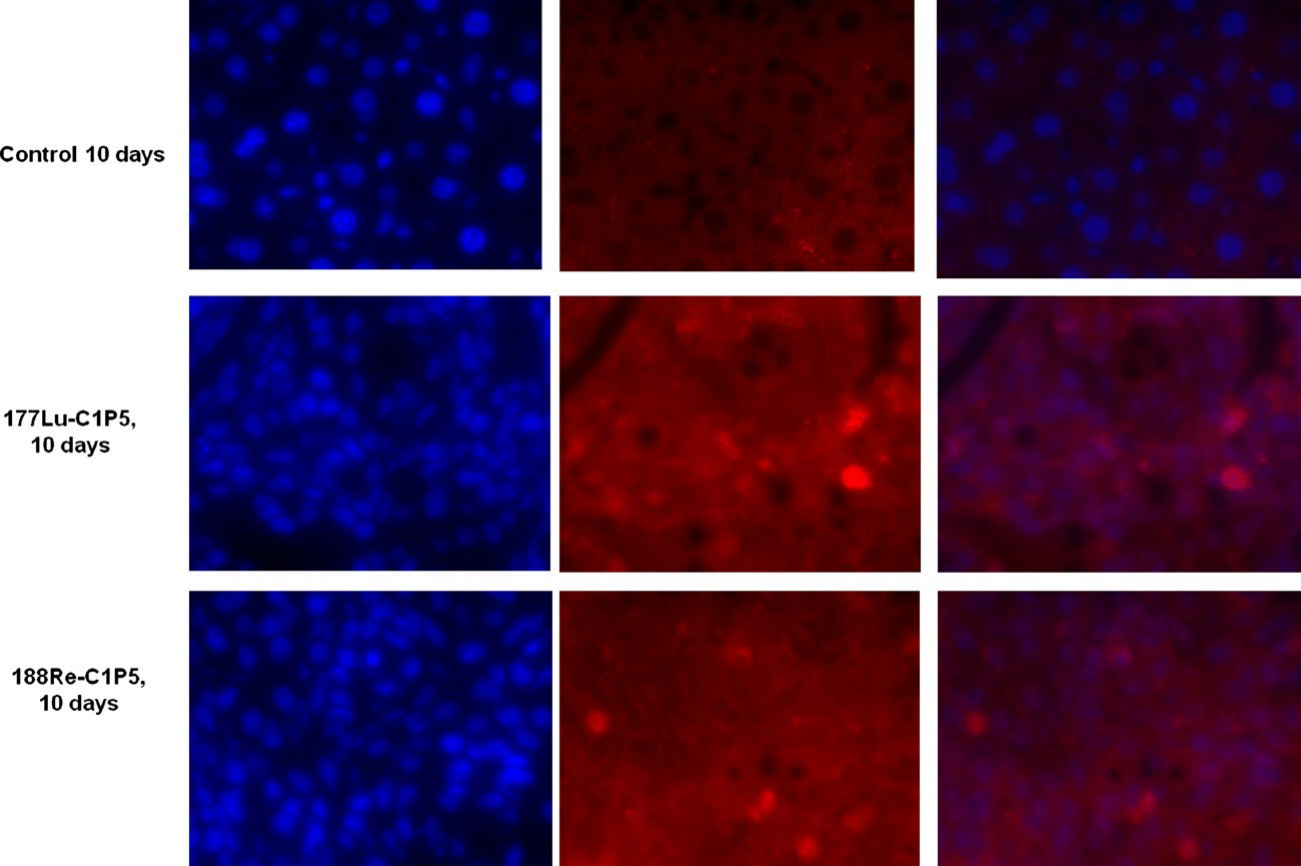
Immunohistochemistry staining for gamma H2AX foci in ^188^Re‐C1P5 and ^177^Lu‐C1P5 tumors on Day 10 post radioimmunotherapy. First row—untreated controls, second row—^177^Lu‐C1P5, third row—^188^Re‐C1P5 treatment. First column—DAPI staining (blue), second column—gamma H2AX foci staining (red), third column—superimposition of DAPI and gamma H2AX staining.

Staining for gamma H2AX foci associated with DSB in the RIT treated tumors on days 5 and 10 posttreatment demonstrated a different timing of the damage to the DNA: on day 5 there was a pronounced staining for gamma H2AX foci in ^177^Lu‐C1P5 treated tumors but not in ^188^Re‐C1P5 treated ones (Fig. 3). On day 10, however, the tumor samples from both ^177^Lu‐C1P5 and ^188^Re‐C1P5 treatment groups exhibited prominent staining for gamma H2AX (Fig. 4).

## Discussion

In our search for effective and safe methods of treating metastatic HPV‐related cancers, such as cervical cancer, we turned to RIT targeting viral antigens because metastatic and recurrent cervical cancer continue to express HPV E6 and E7 oncoproteins. Targeting viral antigens such as HPV‐16/18 E6 oncoprotein has distinct advantages over targeting human “self” antigens because viral antigens are located exclusively on the tumor and nowhere else in the body. Furthermore, the lack of homology between viral and human proteins means that antibodies to viral proteins used in RIT have diminished capacity to cross‐react with human proteins. Both these factors contribute to the exclusive specificity of RIT targeting viral antigens conferring the development of a highly efficacious, minimally toxic treatment modality. In our previous reports, we utilized a high energy beta emitter ^188^Re which proved to be an effective and safe radionuclide for targeting viral antigens 8, 9, 10, 11, 12, 13. However, it is important to know how the different nuclear decay parameters such as penetration range, half‐life, energy of the beta‐emission, and chemistries of alternative to ^188^Re beta emitters would affect the RIT efficacy and its molecular mechanisms. In addition, because commercial availability of radionuclides remains a crucial factor in translating RIT into the clinic 23, it was important for us to utilize a commercially available radionuclide for comparison with ^188^Re. For these reasons we have chosen ^177^Lu as it has its very different from ^188^Re nuclear and chemical properties and performed a comparative RIT study of ^188^Re‐ and ^177^Lu‐labeled C1P5 mAb to HPV16/18 E6 oncoprotein in experimental cervical cancer.

The biodistribution profiles over time of ^188^Re‐ and ^177^Lu‐labeled C1P5 in CasKi tumor‐bearing mice were consistent with the different chemistries of ^188^Re—a transition metal and of ^177^Lu—a radiolanthanide. The higher uptake of ^177^Lu‐C1P5 noted in the tumor and slower clearance from the blood and blood‐rich organs, such as the liver, can be explained by the fact that ^177^Lu, a trivalent metal, preferentially binds to blood proteins such as transferrin. The residualizing nature of ^177^Lu, and, conversely, the fast clearance of ^188^Re from all tissues in form of a chemically inert perrhenate anion was reflected in much higher uptake of ^177^Lu‐C1P5 in the liver than that of ^188^Re‐C1P5 and in its stable uptake in the tumor during the 24–72 h time period post administration of the radiolabeled mAbs. The higher liver and tumor uptake of ^177^Lu‐C1P5 resulted in approximately five times higher dose to the liver and 1.4 times higher dose to the tumor in comparison with ^188^Re‐C1P5. The overall uptake of the radiolabeled mAbs into the tumor was relatively low (~2.5% ID/g) which can be explained by the intranuclear location of the E6 antigen which is only available to the antibody in the dead or nonviable cells within the tumor. In our investigation of other intracellular/intranuclear located antigens such as melanin or single‐strand DNA as potential targets for radiometal‐labeled antibodies we also observed relatively low uptake of the antibodies into the tumors which nevertheless translated into the efficacious therapy in both preclinical and clinical studies 15, 24, 25.

For the RIT study, we chose 7.4 MBq dose for both ^188^Re‐C1P5 and ^177^Lu‐C1P5 mAbs. 7.4 MBq ^188^Re‐C1P5 proved to be effective in slowing down the CasKi tumors growth in our previous studies 10. In spite of relatively low uptake of both antibodies in CasKi tumors observed in biodistribution study, the treatment with either ^188^Re‐C1P5 or ^177^Lu‐C1P5 was very effective in arresting tumor growth in comparison with the untreated controls. As the radiolabeled antibodies take up residence in the tumors, a cascade of multiple cell death mechanisms is activated and set into motion. Thus, the absolute uptake, though low compared to other sites such as liver and blood, confers effective inhibition of tumor growth. This phenomena is similar to treatment effects of external beam radiation therapy 26 where we appreciate that the treatment dose given is not directly proportional to the ongoing therapeutic outcome.

Finally, we compared the ability of ^188^Re‐C1P5 and ^177^Lu‐C1P5 mAbs to cause the DSB in the tumors as indicated by the DSB marker gamma H2AX. At 5 days post‐treatment the DSB caused by ^177^Lu‐C1P5 mAb were very visible in the tumors while almost none were seen in ^188^Re‐C1P5 group. At this particular time point less than one physical half‐life of ^177^Lu had passed and almost 7 half lives—for ^188^Re. The landscape changed at 10 days post‐treatment when both ^188^Re‐C1P5 and ^177^Lu‐C1P5 groups displayed prominent staining for gamma H2AX. By that time all ^188^Re had decayed while only 1.5 half live passed for ^177^Lu. This protraction in inducing DSBs observed for short lived ^188^Re can explain the equal efficacy of ^188^Re‐C1P5 and ^177^Lu‐C1P5 toward tumor growth arrest in spite of 1.4 times higher radiation dose to the tumor for ^177^Lu‐C1P5 than for ^188^Re‐C1P5 mAb. Observation of gamma H2AX staining several days after RIT administration emphasizes the different complex and protracted in time mechanisms involved in RIT effects on the tumor as even bystander cells in RIT experiments exhibit gamma H2AX staining 27 while in the case of external beam radiation, such staining can be observed only within hours post irradiation 28.

In conclusion, we have compared the utility of two beta emitting radionuclides ^188^Re and ^177^Lu that differ in their nuclear and chemical properties for RIT of experimental cervical cancer targeting E6 oncoprotein. We found that ^188^Re‐ and ^177^Lu‐labeled mAbs were equally effective in arresting the growth of CasKi cervical tumors. Thus, both of these radionuclides are candidates for the clinical trial of this approach in patients with advanced, recurrent or metastatic cervical cancer. However, as the dose to the normal liver was more than five times higher for ^177^Lu‐labeled C1P5 mAb than for ^188^Re‐C1P5, it might be advisable to use ^188^Re‐labeled mAb in patients with compromised liver function to avoid additional damage to this organ. The preclinical work in preparation for such trial is currently on‐going.

## Conflict of Interest

None declared.
